# Establishing the Cut-Off Values of 17-Hydroxyprogesterone for Diagnosing Congenital Adrenal Hyperplasia: A Prospective Observational Study in a Tertiary Care Hospital

**DOI:** 10.7759/cureus.76218

**Published:** 2024-12-22

**Authors:** Garima Grover, Rahul Jahagirdar, Ruma Deshpande, Mayur Shinde

**Affiliations:** 1 Pediatrics, Bharati Vidyapeeth (Deemed to be University) Medical College, Pune, IND; 2 Central Research Service, Bharati Vidyapeeth (Deemed to be University) Medical College, Pune, IND

**Keywords:** 17-hydroxyprogesterone, congenital adrenal hyperplasia, incidence, india, newborn screening

## Abstract

Introduction

Congenital adrenal hyperplasia (CAH) is an autosomal recessive disorder primarily caused by 21-hydroxylase enzyme deficiency, impairing cortisol synthesis and resulting in elevated androgen levels. CAH presents in two classical forms: salt-wasting (SW) and simple virilizing (SV). Although CAH is rare in India, regional variations and the absence of a national newborn screening (NBS) program pose significant challenges to accurate diagnosis. This study aimed to assess the incidence of CAH in a regional population and evaluate 17-hydroxyprogesterone (17-OHP) cut-off levels for effective screening.

Methods

This prospective observational study was conducted at a tertiary care hospital in Pune, India, over 18 months (July 2022 to March 2024). Newborns at term with elevated 17-OHP levels in initial screenings were included, while those whose mothers had chronic steroid use were excluded. Heel prick blood samples were collected between 72 and 96 hours postbirth and analyzed using enzyme-linked immunosorbent assay (ELISA). Infants with borderline or elevated initial results underwent a second screening at six weeks, followed by confirmatory testing for adrenocorticotropic hormone (ACTH) and cortisol levels.

Results

Out of 3,607 newborns screened, 638 had elevated 17-OHP levels and were enrolled in the study. The follow-up rate was 36.68%, with 234 infants completing the second screening. Only one infant was diagnosed with CAH, yielding an incidence rate of 1:3,607. This infant presented hyperpigmentation but no atypical genitalia or other classical symptoms of CAH.

Conclusions

The study estimated the incidence of CAH at one in 3,607 live births in our study done in western Maharashtra, India. The findings emphasize the importance of region-specific 17-OHP cut-off values for improving screening accuracy. Establishing a national NBS program is crucial for early detection and timely intervention.

## Introduction

Congenital adrenal hyperplasia (CAH) is an autosomal recessive genetic disorder, most commonly caused by a deficiency in the enzyme 21-hydroxylase (21-OH), which plays a critical role in cortisol synthesis. The disorder presents two main forms: salt-wasting (SW) and simple virilizing (SV). Both forms are characterized by elevated androgen levels due to the enzyme deficiency. In SW CAH, the impairment in enzyme function also affects aldosterone production, resulting in significant salt loss through the kidneys. Without prompt treatment, this can lead to dehydration and shock in neonates. In contrast, SV CAH does not affect aldosterone production, preventing salt loss but still causing virilization. Newborn female neonates with classical CAH may display atypical genitalia, while both sexes can experience progressive virilization after birth. The non-classical form of CAH is milder, presenting no symptoms at birth, but may lead to hyperandrogenism later in life [1,2].

The incidence of CAH in the general population is estimated at one in 13,000 to one in 15,000 live births. In India, the reported incidence is approximately one in 5,762. However, the exact incidence in India remains unclear due to the lack of a national newborn screening (NBS) program for CAH [3-5].

Screening newborns for CAH is recommended because severe forms of the condition can be life-threatening. Early detection and treatment are crucial to ensuring proper growth and development and preventing serious complications. Newborns are typically screened for CAH by measuring 17-hydroxyprogesterone (17-OHP) levels from a dried blood spot obtained via a heel prick. While the goal of these screening programs is to achieve high sensitivity with low false-positive rates, this can be particularly challenging for CAH due to the complex and variable nature of steroid metabolism. Several factors can affect the accuracy of 17-OHP screening, including the age at which the specimen is collected, the newborn's birth weight, gestational age, any existing comorbidities, and the specificity of the assay used [6,7].

A two-tier screening for CAH with repeat measurement of 17-OHP in infants who initially test positive can effectively reduce the number of false positives. Many NBS programs use birth weight and gestational age-related cut-offs to further decrease false positives [8-10]. However, due to significant variations between regional populations, it is advisable for each screening program to establish its reference cut-offs for CAH. Population characteristics can differ widely across different regions, making it essential to standardize 17-OHP reference values for CAH screening within each program [11].

In the absence of a NBS program in India and the prevalent social stigma surrounding atypical genitals at birth, there is a significant lack of data regarding actual incidence and the optimal 17-OHP cut-off values for diagnosing CAH. This study was conducted to assess the incidence of CAH in the population of western Maharashtra, India.

## Materials and methods

This prospective observational study was conducted at the postnatal care unit (PNC) and neonatal intensive care unit (NICU) of a tertiary care hospital in Pune, India, over 18 months from July 2022 to March 2024. The study protocol was reviewed and approved by the institutional ethics committee, ensuring adherence to ethical standards in research involving human subjects. The Bharati Vidyapeeth (Deemed to be University) Medical College Institutional Ethics Committee issued approval BVDUMC/IEC/31.

The study population included all newborns delivered from a term pregnancy or with a corrected gestational age of 37 weeks and having elevated 17-OHP values at the first screening test. Infants whose mothers were on chronic steroid therapy were excluded. Informed consent was obtained from all participants' parents or guardians before their inclusion in the study.

Heel prick samples were collected from all eligible infants between 72 and 96 hours of life, and these samples were analyzed using the enzyme-linked immunosorbent assay (ELISA) method by ELISA reader Bio-Rad PR 4100 (Bio-Rad Laboratories, Hercules, CA, US). Sociodemographic data were collected from the mothers following informed consent and pretest counseling. Clinical features indicative of CAH, such as atypical genitalia, hyperpigmentation, persistent vomiting, failure to thrive, and persistent jaundice, were assessed at three to four weeks to clinically rule out the condition. Infants with borderline or abnormal initial screening results were contacted for a follow-up test after six weeks. Those who had elevated 17-OHP levels on the second screening were subjected to confirmatory tests, including adrenocorticotropic hormone (ACTH) stimulation and cortisol measurement, to definitively diagnose CAH. The 17-OHP values from both the first and second tests were compared with these confirmatory results to determine whether the 17-OHP cut-off levels required revision. ELISA was used for the initial analysis of heel prick samples, and enzyme immunoassay (EIA) was used for the second round of testing for infants with abnormal initial results.

Statistical analysis

Data was coded and entered into a Microsoft Excel spreadsheet (Microsoft Corp., Redmond, WA, US) and analyzed using SPSS version 25.0 (IBM Corp., Armonk, NY, US). Continuous variables were presented as the mean and standard deviation, while categorical variables were presented as frequency and percentage. One-way analysis of variance (ANOVA) was used to assess differences in 17-OHP levels across various groups. A significance level of 5% (95% confidence interval) was used throughout the analysis, with a p-value of less than 0.05 considered statistically significant.

## Results

In this study, 3,607 newborns were screened for eligibility. Among them, 638 infants (17.69%) tested positive on the initial newborn screening and met the inclusion criteria (had values of 17-OHP on the first test above the laboratory cut-off of 35.2 ng/mL) leading to their recruitment into the study. Of these, 234 infants returned for the follow-up second screening test.

Baseline characteristics

Among the 638 neonates included, the most common gestational age was 38 weeks, representing 194 (30.41%) of the total, followed by 37 weeks with 139 (21.79%) and 39 weeks with 135 (21.16%), as shown in Table 1. The mean gestational age was 39.02 weeks with a standard deviation of 1.34 weeks. Regarding birth weight categories, 478 (74.92%) of the neonates were classified as appropriate for gestational age (AGA), while 135 (21.16%) were small for gestational age (SGA), and 25 (3.92%) were large for gestational age (LGA). In terms of gender assigned, 351 (55.02%) were assigned as male neonates and 287 (44.98%) as female neonates. Out of the 638 neonates included in this study, only one had hyperpigmentation of the axilla and gonads. None of the children evaluated had atypical genitalia, persistent vomiting, persistent weight loss after birth, or failure to thrive.

**Table 1:** Baseline characteristics of the study population

Parameter	Value	Frequency (%)
Gestational age (n = 638)	37 weeks	139 (21.79%)
	38 weeks	194 (30.41%)
	39 weeks	135 (21.16%)
	40 weeks	112 (17.55%)
	41 weeks	48 (7.52%)
	42 weeks	10 (1.57%)
	Mean	39.02 weeks
	Standard deviation	1.34 weeks
	Median	38.71 weeks
	Interquartile range (IQR)	2 weeks
Birth weight categories (n = 638)	Appropriate for gestational age (AGA)	478 (74.92%)
	Large for gestational age (LGA)	25 (3.92%)
	Small for gestational age (SGA)	135 (21.16%)
Gender assigned at birth (n = 638)	Female	287 (44.98%)
	Male	351 (55.02%)

First screening

**Figure 1:**
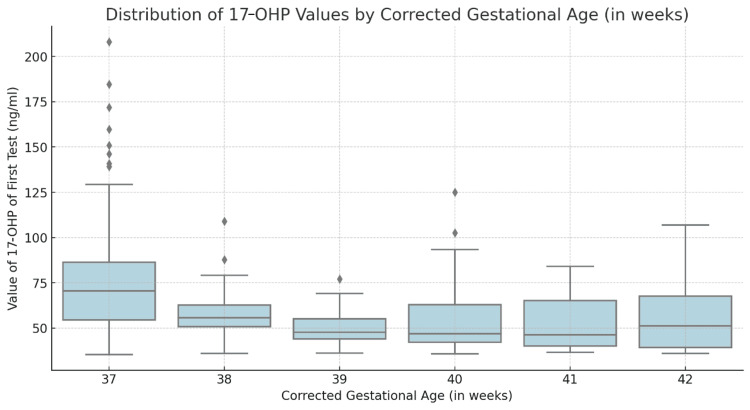
Boxplot graph showing the distribution of the 17-OHP levels at the first screening test according to gestational age 17-OHP: 17-hydroxyprogesterone

The first screening for 17-OHP levels was done at a mean age of 82.80 (+6.53) hours after birth. The mean 17-OHP levels for the neonates included in this study were 58.45 ng/mL (+20.01). As shown in Table 2, the mean 17-OHP levels varied significantly across gestational ages, with neonates born at 37 weeks having the highest mean level of 74.96 ng/mL (±30.56). The levels then decreased as gestational age increased to the lowest mean values of 56.58 ng/mL (±22.04) at 42 weeks (n = 10). The difference in 17-OHP levels across gestational ages was statistically significant, with a p-value of <0.001 (Figure 1).

**Table 2:** Distribution of the mean 17-hydroxyprogesterone (17-OHP) levels on the first screening test Std. Dev.: standard deviation; AGA: appropriate for gestational age; LGA: large for gestational age; SGA: small for gestational age

Parameter	Value	Mean 17-OHP level (ng/mL)	Std. Dev.	Frequency	p-value
Gestational age (weeks)	37	74.96	30.56	139	<0.001
	38	56.87	10.48	194	
	39	49.63	7.72	135	
	40	53.73	16.02	112	
	41	53.12	15.59	48	
	42	56.58	22.04	10	
Assigned gender	Female	58.28	20.2	287	0.849
	Male	58.59	19.88	351	
Birth weight categories	AGA	57.89	20.02	478	0.09
	LGA	54.49	21.22	25	
	SGA	61.15	19.58	135	

When analyzed by gender, the mean 17-OHP level was 58.28 ng/mL (±20.2) in female neonates and 58.59 ng/mL (±19.88) in male neonates, with no significant difference between the two groups (p = 0.849). For birth weight categories, the mean 17-OHP level was 57.89 ng/mL (±20.02) among neonates classified as AGA, 54.49 ng/mL (±21.22) among those classified as LGA, and 61.15 ng/mL (±19.58) among SGA. The variation in 17-OHP levels across these birth weight categories approached statistical significance with a p-value of 0.09.

Second screening

The mean 17-OHP levels for 234 infants who underwent the second test were 7.03 ng/mL (+6.73). Neonates born at 37 weeks had the highest mean 17-OHP level of 8.86 ng/mL (±12.32) among 61 neonates, with levels generally decreasing as gestational age increased, reaching a low of 5.35 ng/mL (±1.56) in those born at 42 weeks (n = 6). However, the differences in 17-OHP levels across gestational ages were not statistically significant (p = 0.2665). When analyzed by gender, male neonates had a slightly higher mean 17-OHP level of 7.55 ng/mL (±8.62) compared to female neonates at 6.36 ng/mL (±2.69), but this difference was also not statistically significant (p = 0.18). Regarding birth weight categories, the mean 17-OHP level was 7.09 ng/mL (±7.75) in AGA neonates, 6.99 ng/mL (±2.73) in SGA neonates, and 5.95 ng/mL (±2.15) in LGA neonates, with no significant differences observed across these groups (p = 0.91) (Table 3).

**Table 3:** Distribution of the mean 17-hydroxyprogesterone (17-OHP) levels on the second screening test Std. Dev.: standard deviation; AGA: appropriate for gestational age; LGA: large for gestational age; SGA: small for gestational age

		Mean 17-OHP level (ng/mL)	Std. Dev.	Frequency	p-value
Gestational age (weeks)	37	8.86	12.32	61	0.2665
	38	6.56	2.76	76	
	39	5.99	2.35	40	
	40	6.59	2.75	44	
	41	6.41	2.5	7	
	42	5.35	1.56	6	
	Total	7.03	6.73	234	
Assigned gender	Female	6.36	2.69	102	0.18
	Male	7.55	8.62	132	
Birth weight categories	AGA	7.09	7.75	169	0.91
	LGA	5.95	2.15	7	
	SGA	6.99	2.73	58	

Out of 234 children screened for a second time, 17-OHP levels were elevated in only one (this baby had 17-OHP of 100 ng/mL in the second screening test). The results of confirmatory tests are given in Table 4. As the total number of neonates screened for this study was 3,607, the estimated incidence of CAH is 1:3,607. This child was assigned male gender at birth with normal genitalia.

**Table 4:** Laboratory values of the solitary case with congenital adrenal hyperplasia (CAH) who was part of this study

Laboratory test	Value	Normal range
17-Hydroxyprogesterone (17-OHP) levels at first screening test	>100 ng/mL	<35.2 ng/mL
17-Hydroxyprogesterone (17-OHP) levels at second screening test	>100 ng/mL	<35.2 ng/mL
Adrenocorticotropic hormone (ACTH)	201 pg/mL	7.2-63 pg/mL
Cortisol	4.7 mcg/dL	3.70-19.4 mcg/dL

## Discussion

The present study conducted newborn screening for CAH by measuring 17-OHP levels in neonates at a hospital in Pune, India. Out of 3,607 neonates screened, 638 were included, and 234 appeared for a follow-up screening after six weeks. Elevated 17-OHP levels were found in one child who was later diagnosed with CAH based on confirmatory tests for cortisol and ACTH levels.

Overall, based on the findings of this research, the incidence of CAH was estimated to be 1:3,607. However, this may not reflect the true incidence, as the second test could not be done in more than half of the neonates that were eligible for it. Previously done research has also reported variable incidence of CAH among the Indian population.

In a study done at multiple centers, significant variations were observed in the incidence of CAH across India, being 1:2,036 in Chennai, 1:7,608 in Delhi, and 1:9,983 in Mumbai. Similarly, Kumar et al. reported the incidence of CAH as 1:2,800 in a South Indian population, while Kaur et al. reported it to be 1:6,334 in neonates from North India. The study conducted by Nordenström et al. at the Karolinska Institute in Stockholm, Sweden, and the Royal Manchester Children’s Hospital and St. Mary’s Hospital in Manchester, UK, reported the incidence of CAH to be 6.17 cases per 100,000 live births [12]. The varying rates of CAH reported in India may be attributed to differences in thresholds and/or the screening of different populations attributed to genetic factors, environmental factors, and laboratory values. The higher incidence in the Indian population could be attributed to the higher rates of consanguinity, which increases the risk of transmission of genetic disorders [13,14].

Research indicates that when the sample is collected, it has a significant impact on 17-OHP levels, which are highest right after birth and then decrease in the first few days of life. For example, a study by Gidlöf et al. in Sweden showed that the best time for screening is between 48 and 72 hours after birth to achieve a balance between sensitivity and specificity [6]. Our study revealed that the average age at the initial screening was 82.80 hours (approximately 3.5 days) after birth, which corresponds with the recommended timing observed in these studies. This timing helps reduce the impact of the immediate surge in 17-OHP levels after birth, leading to more accurate screening results.

The level of 17-OHP in newborns is significantly influenced by the gestational age at birth, which affects the accuracy of CAH screening. Hayashi et al. found that modifying 17-OHP threshold values according to gestational age resulted in a notable enhancement of screening specificity and a decrease in recall rates [8]. In our study, we only included neonates delivered at full term with a mean gestational age of 39.02 weeks who had elevated 17-OHP on the first test. Despite this, the results from the first screening showed a significant decline in 17-OHP levels with increasing gestational age (p = 0.001). This difference in 17-OHP levels disappeared after six weeks when the second screening test was done.

In our study, we found that the average levels of 17-OHP were similar in neonates, regardless of their assigned gender at birth (p = 0.849). The mean levels were 58.28 ng/mL in female neonates and 58.59 ng/mL in male neonates during the first screening, and 6.36 ng/mL in female neonates and 7.55 ng/mL in male neonates during the second screening (p = 0.18). For instance, Nordenström et al. discovered that male neonates exhibited slightly elevated levels of 17-OHP in comparison to female neonates, possibly attributed to variances in adrenal function [12]. However, Gidlöf et al. found that there was no notable difference in 17-OHP levels between male and female neonates, underscoring the importance of establishing gender-neutral threshold values for efficient screening [6]. This consistency across different studies reinforces the reliability of our screening protocol and highlights the negligible impact of gender on 17-OHP levels in the context of CAH screening.

The link between birth weight and 17-OHP levels in newborns is crucial for accurately screening for CAH. In our research, the average 17-OHP levels during the initial screening were 61.15 ng/mL for SGA, 57.89 ng/mL for AGA, and 54.49 ng/mL for LGA newborns, and no significant variances were observed among these groups (p = 0.09). During the second screening, the average 17-OHP levels were 6.99 ng/mL for SGA, 7.09 ng/mL for AGA, and 5.95 ng/mL for LGA newborns, again showing no significant differences (p = 0.91). These outcomes are consistent with van der Kamp et al.'s findings, which suggested that while there might be minor fluctuations in 17-OHP levels based on birth weight, the variances are not statistically significant enough to warrant separate thresholds in our cohort [15].

Limitations

Despite the significant findings, this study has several limitations that should be acknowledged. The sample was restricted to a single geographic region in Maharashtra, India, which may limit the generalizability of the results to other regions with different population characteristics. Additionally, the study included only full-term neonates, excluding preterm infants who are known to have higher and more variable 17-OHP levels, potentially missing critical insights relevant to this subgroup. Although the study used a robust sample size, the follow-up rate for the second screening was lower than desired, with only 234 out of 638 initially screened neonates returning for the follow-up test. This attrition could have introduced bias, affecting the reliability of the second screening results. The main reason for the same is that the families went to their native places and villages after the delivery. They were contacted telephonically for any clinical features and advised to follow up for the second test. However, low follow-up was attributed to factors like transportation issues, poor awareness, and social stigma associated with hormonal disorders.

Furthermore, the study did not explore the potential impact of various maternal and perinatal factors, such as maternal health, medications, and delivery complications, which could influence 17-OHP levels in newborns. The absence of a NBS program in India also means that the findings are based on a non-standardized approach, which could differ from internationally established protocols. Moreover, the estimation of the optimal cut-off value of 17-OHP could not be fully realized due to only one confirmed case of CAH in the study, which limited the ability to perform a detailed statistical analysis. Future studies should aim to address these limitations by including a more diverse and larger sample size, accounting for preterm infants, and considering additional maternal and perinatal factors to enhance the comprehensiveness and applicability of the findings.

## Conclusions

In conclusion, this study provides valuable insights into the screening for CAH using 17-OHP levels in a regional population in Maharashtra, India. The findings suggest that while the timing of the initial screening, conducted at approximately 82.80 hours after birth, aligns with recommended practices and may improve the accuracy of CAH detection, several challenges remain. The study identified an estimated incidence of CAH at one in 3,607 neonates. This study underscores the importance of establishing accurate, region-specific cut-off values for 17-OHP in newborn screening for CAH. Using gestational age-specific cut-offs can improve detection accuracy, reduce false positives, and ensure timely intervention.
